# Menstrual Tracking Mobile App Review by Consumers and Health Care Providers: Quality Evaluations Study

**DOI:** 10.2196/40921

**Published:** 2023-03-01

**Authors:** Siyeon Ko, Jisan Lee, Doyeon An, Hyekyung Woo

**Affiliations:** 1 Department of Health Administration College of Nursing & Health Kongju National University Gongju-Si, Chungcheongnam-do Republic of Korea; 2 Department of Nursing Science College of Life & Health Sciences Hoseo University Asan-si, Chungcheongnam-do Republic of Korea; 3 Department of Nursing Gangneung-Wonju National University Wonju-si Republic of Korea; 4 Institute of Health and Environment Kongju National University Gongju-si, Chungcheongnam-do Republic of Korea

**Keywords:** mobile app, period, menstrual cycle, mHealth, mobile health, evaluation, women’s health, health care provider, consumer, menstrual app, digital health app, health screening, consumer satisfaction

## Abstract

**Background:**

Women’s menstrual cycle is an important component of their overall health. Physiological cycles and associated symptoms can be monitored continuously and used as indicators in various fields. Menstrual apps are accessible and can be used to promote overall female health. However, no study has evaluated these apps’ functionality from both consumers’ and health care providers’ perspectives. As such, the evidence indicating whether the menstrual apps available on the market provide user satisfaction is insufficient.

**Objective:**

This study was performed to investigate the key content and quality of menstrual apps from the perspectives of health care providers and consumers. We also analyzed the correlations between health care provider and consumer evaluation scores. On the basis of this analysis, we offer technical and policy recommendations that could increase the usability and convenience of future app.

**Methods:**

We searched the Google Play Store and iOS App Store using the keywords “period” and “menstrual cycle” in English and Korean and identified relevant apps. An app that met the following inclusion criteria was selected as a research app: nonduplicate; with >10,000 reviews; last updated ≤180 days ago; relevant to this topic; written in Korean or English; available free of charge; and currently operational. App quality was evaluated by 6 consumers and 4 health care providers using Mobile Application Rating Scale (MARS) and user version of the Mobile Application Rating Scale (uMARS). We then analyzed the correlations among MARS scores, uMARS scores, star ratings, and the number of reviews.

**Results:**

Of the 34 apps, 31 (91%) apps could be used to predict the menstrual cycle, and 2 (6%) apps provided information pertinent to health screening. All apps that scored highly in the MARS evaluation offer a symptom logging function and provide the user with personalized notifications. The “Bom Calendar” app had the highest MARS (4.51) and uMARS (4.23) scores. The MARS (2.22) and uMARS (4.15) scores for the “Menstrual calendar—ovulation & pregnancy calendar” app were different. In addition, there was no relationship between MARS and uMARS scores (*r*=0.32; *P*=.06).

**Conclusions:**

We compared consumer and health care provider ratings for menstrual apps. Continuous monitoring of app quality from consumer and health care provider perspectives is necessary to guide their development and update content.

## Introduction

### Background

Women’s menstrual cycles are important to their overall health [1,2] and are characterized by predictable and recurring symptoms. Continuous tracking of the menstrual cycle can aid in health management. Monitoring systems are needed to optimize menstrual health and provide easily accessible health information for women [3].

Mobile health (mHealth) apps facilitate personalized health monitoring and management [4], and menstrual apps are the most important mHealth apps for women. Such apps are typically highly accessible for most women and provide indicators relevant to various health domains [5].

Typically, menstrual apps also allow the user to log their symptoms, mood changes, and body temperature, and they visually represent statistical data via graphs and tables. Some apps offer professional-level information through communities and links, that is, they promote women’s health care by facilitating smooth communication with medical staff through information-sharing services [6]. The apps’ menstrual cycle tracking functions facilitate health care planning and management in various domains, including contraception, fertility, preparation with respect to pregnancy and ensuring adequate “menstrual supplies,” leisure activities, and travel [7].

Women use these functions for various purposes, such as tracking pregnancy, preventing pregnancy, and managing menstruation periods [8]. The functions desired by consumers depend on the intended purpose of the app. Currently, indicators to help consumers identify and select menstrual apps according to the desired functions are lacking [9]. Recently developed systems recommend apps based on consumer requirements [10,11]. Menstrual apps with various functions have been developed, and related research is being actively conducted. However, most of the existing studies are content reviews or expert evaluations [12-15]. Consumer-centered studies have also started to appear [5,16-18], but quality evaluations of menstrual apps remain scarce.

Menstrual apps are directly relevant to women’s health, so it is necessary for experts and health care providers to evaluate these apps [8]. Health care provider quality evaluations can contribute to the app development, which is important to ensure that consumers have access to high-quality apps [19]. Consumer quality evaluations provide feedback on apps, such as consumer preferences (eg, for easy-to-use content) [20,21]. Consistent use of an app is critical given the recurring nature of the menstrual cycle, but research on this topic is lacking from the developer’s standpoint. To promote sustained app use, evaluations from consumers and health care providers’ perspective are required. However, it is unclear whether current commercially available apps satisfy the quality standards of consumers and health care providers. If we find the differences between consumers and health care providers, they need to be discussed importantly to develop apps that can be used to promote women’s health that satisfy both consumers’ wants and health care providers’ needs.

### Purpose

This study examines currently available menstrual apps in term of their main contents and quality based on evaluations by health care providers and consumers. We also correlated the two sets of evaluation scores. The study’s findings could improve the utility and convenience of future mHealth apps.

## Methods

### App Selection

We searched for keywords related to the development and evaluation of menstrual apps commonly included in previous studies, such as “period” and “menstrual cycle” in both English and Korean. The Google Play Store and the iOS App Store were searched from April 8 to April 15, 2021. Up to 150 apps were screened for each keyword. Since then, to secure the appropriated number of apps that can be statistically analyzed, the following app inclusion criteria have been set, based on previous studies [14,22,23]:

- The app is nonduplicate.
- It has more than 10,000 reviews.
- It has been last updated ≤180 days ago.
- It is relevant to this topic.
- The app language is Korean or English.
- The app is free to use.
- It is currently in operation.

### Analysis of App Contents

To examine the apps’ main contents, we performed a pilot study of 14 representative apps. The apps’ main contents were classified as follows: menstrual cycle management, education and knowledge, sharing information, and notifications. Frequency analysis was performed to determine the number of apps providing these functions.

### Evaluation of App Quality

The quality of 34 menstrual apps were evaluated using Mobile Application Rating Scale (MARS) and user version of the Mobile Application Rating Scale (uMARS). The MARS was developed to evaluate mobile apps; it is a reliable tool with a high internal consistency and interrater reliability [24]. The uMARS was subsequently developed, based on the MARS, to allow consumers to evaluate the quality of mHealth apps. uMARS has excellent internal consistency [25]. Both scales comprise the following five categories: engagement, aesthetics, functionality, information, and subjective quality (Table 1). The MARS has been used to evaluate various health care–related apps, such as apps related to chronic disease, COVID-19, and physical activity, as well as apps related to allergy, hepatitis treatment support, and breast cancer [14,15,19,22,23,26,27]. The uMARS has recently been used to evaluate various types of mHealth apps, including apps pertaining to weight loss and nutrition, rheumatic diseases, and the management of ankylosing spondylitis [28-31].

**Table 1:** Mobile Application Rating Scale (MARS) and user version of the Mobile Application Rating Scale (uMARS) evaluation items.

Measures		Characteristics
**Objective measures**		
	Engagement	Entertainment^a,b^ Interest^a,b^ Customization^a,b^ Interactivity^a,b^ Target group^a,b^
	Functionality	Performance^a,b^ Ease of use^a,b^ Navigation^a,b^ Gestural design^a,b^
	Information	Accuracy of app design^a^ Goal^a^ Credibility^a^ Evidence bases^a^ Quality of information^a,b^ Quantity of information^a,b^ Visual information^a,b^ Credibility of source^b^
	Aesthetics	Layout^a,b^ Graphics^a,b^ Visual appeal^a,b^
**Subjective measures**		
	Subjective quality	Recommendation^a,b^ Frequency of use^a,b^ Payment for expenses^a,b^ Star rating^a,b^

^a^MARS evaluation items.

^b^uMARS evaluation items.

A total of 6 consumers completed the uMARS between July 9 and July 22, 2021, and 4 nurses (ie, health care providers) majoring in health care and working in medical centers completed the MARS between July 21 and July 30, 2021. Each evaluator was asked to use the app for more than 10 minutes every day and the evaluation was conducted in a blind test. The apps were randomly assigned to evaluators to prevent bias related to subjectivity. Each app was crossevaluated by at least two evaluators.

### Comparative Analysis of Consumer and Health Care Provider Data

The MARS results represent health care providers’ perspectives, as stated above. The uMARS results, star ratings, and number of reviews were analyzed from the consumers’ perspective. After normalization, Pearson correlation was used to correlate app content, MARS and uMARS scores, star ratings, and reviews and to correlate perspectives of health care providers and consumers. We identified the top and bottom five apps based on the MARS and uMARS scores and compared app preferences between consumers and health care providers. *P* values <.05 were considered significant. R software (version 4.1.2; R Core Team,) was used for the analysis.

## Results

### App Selection

**Figure 1:**
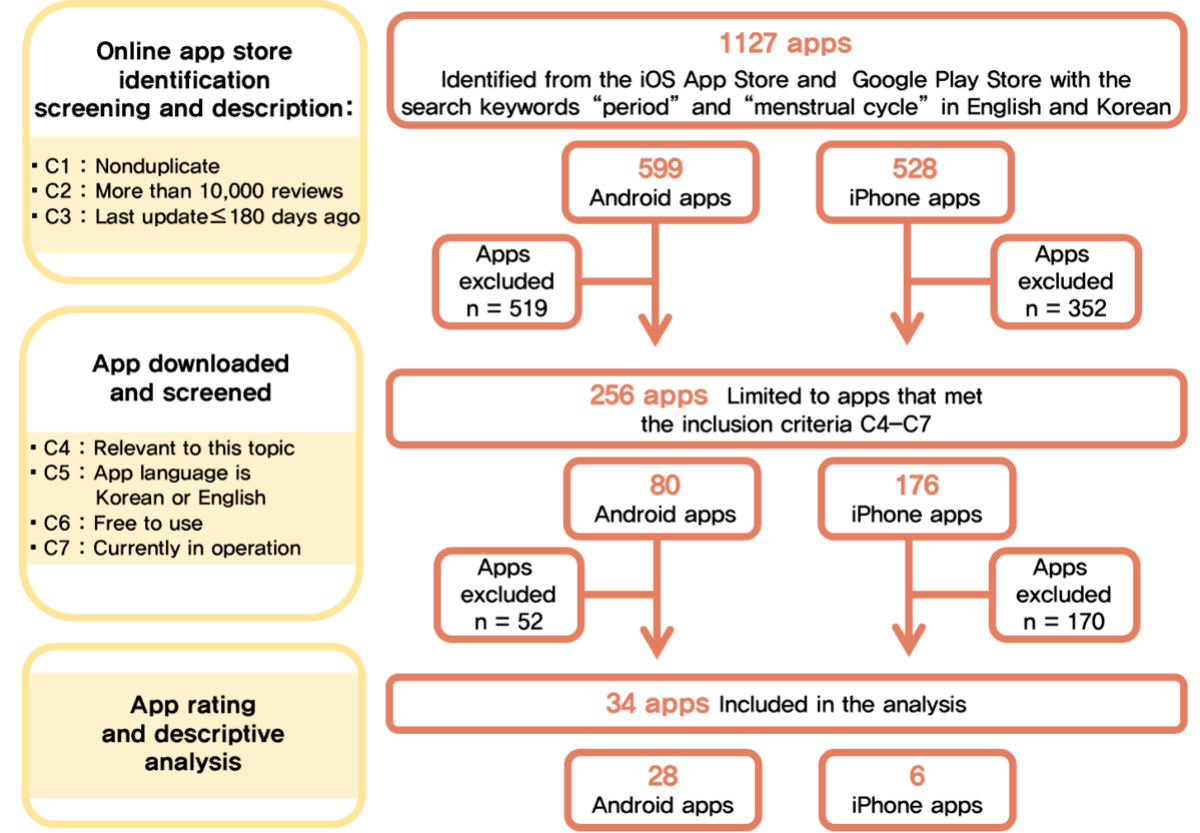
Flow diagram of the app review and selection process. C1-7 denotes Criterion 1-7.

A total of 1127 menstrual apps were initially identified via the keyword search, and 34 apps (Android: n=28; iPhone: n=6) met all of the study criteria and were included in the final analysis (Figure 1).

The operating system (OS) of apps is linked with the app store, so that the app is updated simultaneously with updates of the OS [32]. Therefore, the app version and function may vary depending on the timing of the OS update. The number of reviews and star ratings differed among the apps in this study. For example, Bom Calendar had star ratings of 4.8 and 4.4 for the Android and iOS versions, respectively (23,437 and 76,258 reviews, respectively). In instances where the same apps were available for different OSs, each version was considered to be a unique app in the analysis.

### Analysis of App Contents

Most apps (n=31, 91%) offered a menstrual cycle prediction function. Some apps (n=14, 41%) offered menstruation and fertility period notifications, while others had no specific functions (n=3, 9%). Most apps were confidential (n=29, 85%), allowed data export (n=28, 82%), and had a log-in function (n=25, 74%). However, few apps provided education or knowledge (n=10, 29%), screening-related information (n=2, 6%), or advice (n=4, 12%; Table 2).

**Table 2:** App contents analysis result.

Contents			App, n (%)			
			Android (n=28)		iPhone (n=6)	Total (N=34)
**Menstrual cycle management**						
	Symptoms (pain)	4 (14)		0 (0)		4 (12)
	Additional symptom	18 (64)		6 (100)		24 (71)
**Ovulation management**						
	Calculate pregnancy probability	4 (14)		0 (0)		4 (12)
	Contraception methods	3 (11)		0 (0)		3 (9)
	Both	12 (43)		6 (100)		18 (53)
**Last update (months)**						
	<3	21 (75)		5 (83)		26 (76)
	3~6	7 (25)		1 (17)		8 (24)
**Function** **s**						
	Graphical chart	17 (61)		4 (67)		21 (62)
	Lock	23 (82)		6 (100)		29 (85)
	Advice provision	2 (7)		2 (33)		4 (12)
	Data export	23 (82)		5 (83)		28 (82)
	Predictions	25 (89)		6 (100)		31 (91)
	Log-in	20 (71)		5 (83)		25 (74)
**Education or knowledge**						
	General health information	5 (18)		1 (17)		6 (18)
	Personalized information	1 (4)		2 (33)		3 (9)
	Both	1 (4)		0 (0)		1 (3)
	Health screening	1 (4)		1 (17)		2 (6)
**Sharing information (with health care professionals)**						
	All information	4 (14)		3 (50)		7 (21)
	Only information specified by the consumer	1 (4)		0 (0)		1 (3)
**Visualization**						
	Menstruation or ovulation	2 (7)		0 (0)		2 (6)
	Menstrual cycle	9 (32)		0 (0)		9 (26)
	All data	16 (57)		6 (100)		22 (65)
**Notifications**						
	Menstruation or fertility	2 (7)		0 (0)		2 (56)
	Both	14 (50)		0 (0)		14 (41)
	Personalized alarms	9 (32)		6 (100)		15 (44)
**Other features**						
	Community	4 (14)		2 (33)		6 (18)
	Shopping	2 (7)		1 (17)		3 (9)

### Evaluation of App Quality

The MARS and uMARS scores of all apps were obtained through the average of the evaluator’s evaluation scores. The average MARS and uMARS scores (ie, health care provider and consumer scores, respectively) were 3.06 (SD 0.62) and 3.33 (SD 0.57), respectively. The iPhone “Bom Calendar” app had the highest score for both the MARS (4.51, SD 0.22) and uMARS (4.23, SD 0.27). The Android “Period calendar—Women’s menstrual calendar❤” app had the second lowest score for both the MARS (2.05, SD 0.45) and uMARS (2.09, SD 0.05), despite its high star rating of 4.8. The Android “Menstrual calendar—ovulation & pregnancy calendar” app showed contrasting scores between the MARS (2.22, SD 0.07) and uMARS (4.15, SD 0.46); it had the third highest overall uMARS score, with an engagement score of 3.86 (SD 0.49), functionality score of 4.04 (SD 0.49), aesthetics score of 4.25 (SD 0.47), information score of 4.25 (SD 0.47), and subjective quality score of 4.37 (SD 0.41). However, for the MARS, its overall score was the third lowest, with an engagement score of 2.40 (SD 0.20), functionality score of 2.50 (SD 0.50), aesthetics score of 2.00 (SD 0.00), information score of 2.58 (SD 0.42), and subjective quality score of 1.63 (SD 0.38; Table 3 and Table 4). MARS and uMARS scores of all 34 apps are presented in Multimedia Appendix 1.

**Table 3:** The five highest- and lowest-scoring apps (N=34) based on the user version of the Mobile Application Rating Scale (uMARS). All the values are mean (SD).

App			Engagement		Functionality		Aesthetics		Information		Subjective quality		uMARS^a^
**Top five^b^**													
	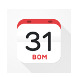	3.87 (0.33)		4.17 (0.26)		4.33 (0.32)		4.27 (0.29)		4.51 (0.22)		4.23 (0.27)	
	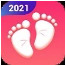	4.03 (0.38)		4.16 (0.44)		4.23 (0.35)		4.31 (0.27)		4.40 (0.25)		4.23 (0.33)	
	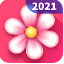	3.86 (0.49)		4.04 (0.49)		4.25 (0.47)		4.25 (0.47)		4.37 (0.41)		4.15 (0.46)	
	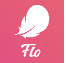	3.80 (1.01)		3.97 (0.78)		4.30 (0.51)		4.30 (0.51)		4.29 (0.49)		4.13 (0.66)	
	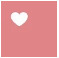	3.83 (0.60)		4.06 (0.43)		4.16 (0.30)		4.11 (0.35)		4.39 (0.36)		4.11 (0.38)	
**Bottom five^c^**													
	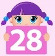	1.91 (0.34)		1.96 (0.39)		1.91 (0.54)		1.94 (0.75)		1.90 (0.72)		1.93 (0.54)	
	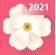	1.76 (0.11)		2.04 (0.13)		2.17 (0.07)		2.13 (0.10)		2.33 (0.10)		2.09 (0.05)	
	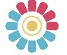	1.93 (0.38)		2.11 (0.53)		2.46 (0.47)		2.53 (0.37)		2.46 (0.51)		2.30 (0.44)	
	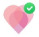	2.30 (0.30)		2.50 (0.50)		2.70 (0.30)		2.74 (0.16)		2.90 (0.10)		2.63 (0.23)	
	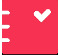	2.24 (1.05)		2.43 (1.24)		3.06 (1.07)		3.19 (1.19)		3.33 (1.33)		2.85 (1.17)	

^a^Average of uMARS scores evaluated by consumers.

^b^Top five apps: (1) Bom Calendar (iOS), (2) Pregnancy planning & management (Android), (3) Menstrual calendar—ovulation & pregnancy calendar (Android), (4) Flo (Android), and (5) Flo (iOS).

^c^Bottom five apps: (1) Maya—My Period Tracker (Android); (2) Period calendar—Women’s menstrual calendar❤ (Android); (3) Women’s menstrual calendar, menstrual & ovulation day calculator, childbearing age, pregnancy planning (Android); (4) My Days—Ovulation Calendar & period Tracker (Android); and (5) Clover: Period & Cycle Tracker (Android).

**Table 4:** The five highest- and lowest-scoring apps (N=34) based on the Mobile Application Rating Scale (MARS).

App and operating system			Engagement		Functionality		Aesthetics		Information		Subjective quality		MARS^a^
**Top five^b^**													
	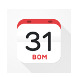	4.18 (0.63)		4.75 (0.25)		5.00 (0.00)		4.29 (0.11)		4.36 (0.25)		4.51 (0.14)	
	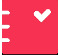	4.10 (0.10)		4.06 (0.21)		4.67 (0.33)		4.92 (0.08)		3.93 (0.24)		4.34 (0.15)	
	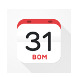	3.85 (0.46)		4.13 (0.63)		4.42 (0.43)		4.00 (0.33)		3.58 (0.58)		3.99 (0.48)	
	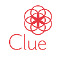	3.65 (0.75)		4.19 (0.57)		4.00 (1.00)		4.42 (0.58)		3.68 (0.07)		3.99 (0.59)	
	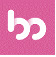	4.00 (0.00)		3.56 (0.37)		4.83 (0.17)		4.33 (0.35)		3.02 (0.61)		3.95 (0.29)	
**Bottom five^c^**													
	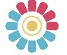	2.05 (0.65)		2.13 (0.63)		1.50 (0.50)		2.38 (0.71)		1.53 (0.53)		1.92 (0.60)	
	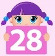	2.10 (0.71)		2.81 (0.48)		2.08 (0.60)		2.25 (0.25)		1.01 (0.26)		2.05 (0.45)	
	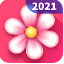	2.40 (0.20)		2.50 (0.50)		2.00 (0.00)		2.58 (0.42)		1.63 (0.38)		2.22 (0.07)	
	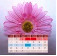	2.00 (0.20)		2.94 (0.57)		2.42 (0.43)		2.63 (0.14)		1.34 (0.09)		2.26 (0.15)	
	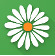	2.00 (0.00)		2.75 (0.25)		2.33 (0.33)		2.75 (0.08)		1.81 (0.56)		2.33 (0.01)	

^a^Average of MARS scores evaluated by health care providers.

^b^Top five apps: (1) Bom Calendar (iOS), (2) Pink Diary (Android), (3) Bom Calendar (Android), (4) Clue Period, Ovulation Tracker (iOS), and (5) Femometer—Fertility Tracker (Android).

^c^Bottom five apps: (1) My Days—Ovulation Calendar & period Tracker (Android), (2) Period calendar—Women’s menstrual calendar❤ (Android), (3) Menstrual calendar—ovulation & pregnancy calendar (Android), (4) My Menstrual Diary (Android), and (5) Period Tracker (Android).

### Further Comparative Analysis of Consumer and Health Care Provider Data

Table 5 shows the results of correlation analysis of the MARS and uMARS scores, star ratings, number of reviews, and app content. The number of reviews was not correlated with app content and menstrual cycle management (*r*=0.53; *P*=.001), and visualization (*r*=0.51; *P*=.002) had the highest correlation with star ratings. Among the evaluation scores, the highest correlation was found between uMARS and notification (*r*=0.39; *P*=.02) as well as between MARS and ovulation date management (*r*=0.49; *P*=.003).

Multimedia Appendix 2 shows content comparison between the top and bottom five apps in consumer and health care provider evaluation scores. Personalized alarms could be set in the top five apps. In addition, they provided a function to visualize all information through a calendar or to specify and manage ovulation days. On the contrary, in the bottom five apps did not have functions for managing or predicting the menstrual cycle.

**Figure 2:**
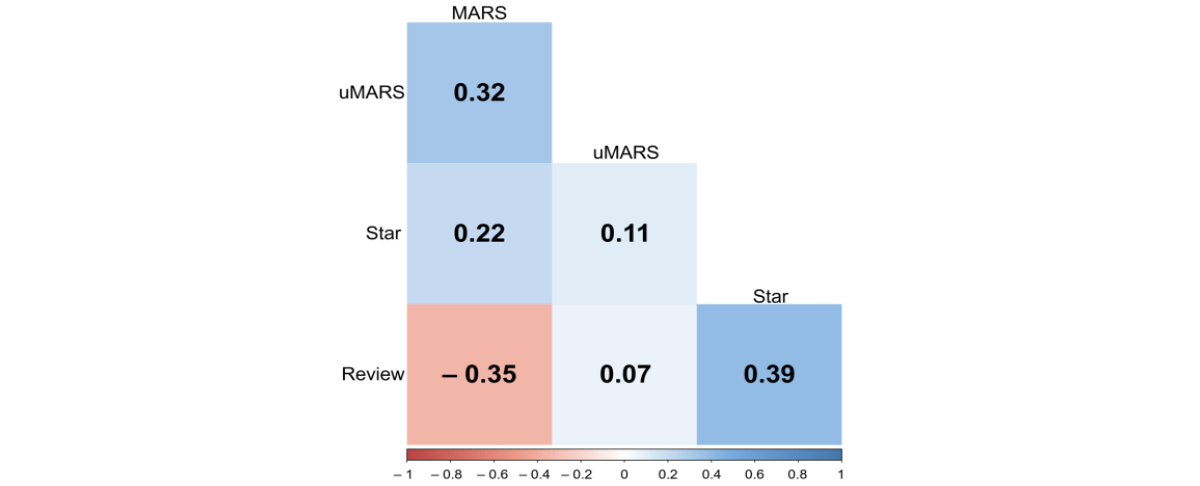
Result of the correlation analysis of the Mobile Application Rating Scale (MARS) and user version of the Mobile Application Rating Scale (uMARS) scores, star ratings, and number of reviews.

Figure 2 shows the correlations among the MARS and uMARS scores, star ratings, and number of reviews to compare the perspective of health care providers and consumers. Figure 2 shows no correlation between MARS and uMARS scores of the health care providers and consumers (*r*=0.32; *P*=.06). uMARS scores and star rating (*r*=0.11; *P*=.54) as well as uMRAS scores and number of reviews (*r*=0.07; *P*=.67) also showed no significant correlations. The number of reviews and star rating (*r*=0.39; *P*=.02) showed a very low correlation.

**Table 5:** Results of correlation analysis of the Mobile Application Rating Scale (MARS) and the user version of the Mobile Application Rating Scale (uMARS) scores, star rating, number of reviews, and app content types.

App contents		Health care provider and consumer perspective			
		MARS	uMARS	Star rating	Number of reviews
Menstrual cycle management		0.39^a^	0.18	0.53^b^	0.21
Ovulation date management		0.49^b^	0.33	0.41^a^	0.16
**Functions**					
	Graphic chart	–0.06	–0.10	–0.54^b^	–0.31
	Lock	–0.37^a^	–0.40^a^	–0.32	–0.10
	Advice provision	–0.05	–0.31	–0.25	–0.01
	Data export	–0.22	–0.20	–0.53^b^	–0.10
	Prediction	–0.43^a^	–0.17	–0.79^b^	–0.22
	Log-in	–0.12	–0.01	–0.47^b^	–0.13
Educational or knowledge		0.23	0.04	–0.02	–0.14
Health screening		0.25	0.13	0.12	0.01
Sharing information		0.16	0.14	0.04	–0.13
Visualization		0.32	0.22	0.51^b^	0.19
Notification		0.32	0.39^a^	0.24	0.06
**Other features**					
	Community	–0.14	–0.19	–0.16	–0.10
	Shopping	–0.35^a^	–0.05	–0.14	0.12

^a^*P*<.05.

^b^*P*<.01.

## Discussion

### Principal Findings and Comparison With Prior Work

Interest in mHealth apps has recently increased, and new apps are continuously being developed, including menstrual apps [33]. However, the needs of consumers and health care providers are different, and studies evaluating whether the available menstrual apps satisfy both of these groups are difficult to find. This study obtained quality evaluations of relevant apps from consumers and health care providers using the uMARS and MARS, respectively; the consistency of the evaluations of these two groups in terms of key app contents was analyzed. The health care providers valued engagement, functionality, and aesthetics when evaluating apps, while consumers valued aesthetics and information provision the most.

The MARS and uMARS scores were not correlated in this study. For example, the app with the third lowest MARS score had the third highest uMARS score. A significant difference was observed in app aesthetics scores between the consumers and health care providers; the scores for this attribute showed the largest group difference. Previous studies on health services have reported disparities between consumers and health care providers, and these results affect the implementation of consumer-centered services. Data from health care providers can provide a basis for high-quality apps [20], while consumer data serves as feedback on app quality [19]. To ensure high app quality and consumer satisfaction, app quality should be continuously monitored from the perspective of both health care providers and consumers. Monitoring can identify the needs of consumers and health care providers, which can in turn help in app development and update [10]. This study focused on evaluating app quality using MARS and uMARS for consistency, but could be extended to qualitative studies, including interviews, to collect in-depth answers in the future [34].

According to our findings, the uMARS (ie, consumer) scores, star ratings, and number of reviews were unrelated variables. The uMARS allows for direct assessment of mHealth apps and is a reliable measure of app quality [25]. However, reviews and star ratings are subjective indicators [24]. The currently available mHealth apps have been evaluated in a simplistic manner, such as through star ratings and reviews, even though they differ significantly with respect to content; thus, appropriate guidelines to aid app selection are lacking. By using the uMARS to guide app selection, the limitations of reviews and star ratings can be overcome such that consumers will likely select more useful apps to meet their particular needs. However, it is difficult for consumers to evaluate the quality of the apps using uMARS every time they download one. New indicators to guide app selection are needed so that consumers can make decisions based on objective evaluation results.

Most of the five apps that scored highly in the quality evaluation in this study included personalized monitoring functions. Menstrual apps are becoming increasingly popular, and apps that include self-monitoring functions and provide related information are continuously being developed [28]. Personalized monitoring can improve user well-being by encouraging them to check for signs and symptoms of health issues. Health-related information can also promote consumer health. mHealth apps that include personalized content of this nature are particularly useful for consumers [23,35]. However, only a few of the apps evaluated in this study facilitated consultations with specialists or provided information relating to women’s health. To increase the utility of apps, notifications, symptom recording functions, and the provision of knowledge should be prioritized.

mHealth apps should provide customized content for individual consumers [36]. However, the five bottom-scoring apps in this study did not meet the needs of the women who were using them. Consumers use apps to predict menstrual cycles and ovulation dates, and to monitor their general health [37]. However, most of the five bottom-scoring apps did not provide content enhancing consumer convenience, such as functions for menstrual cycle and ovulation day management, and some apps also lacked predictive functions. Such apps must be updated to include content allowing for the prediction and management of menstrual cycles based on accumulated menstrual cycle- and health-related information.

Menstrual apps collects personal information from consumers, such as name, date of birth, menstrual cycle, and medical history [38]. Personal data must be protected because it is sensitive information [12,39], but some apps do not provide locking functions, and few apps provide icon change functions for protecting personal information. Most fitness apps that record the number of steps do not consider privacy issues, and the data protection of mHealth apps related to women’s health is typically poor [39]. Therefore, regulations pertaining to app management of private data are necessary [40,41]. In fact, there are existing regulations protecting personal information, such as the European Unions’s General Data Protection Regulation, but no standard regulations are enforced worldwide. mHealth apps that protect personal information tend to be favored by consumers [21]. App developers should improve data protection–related functions to protect the personal information of consumers.

The MARS and uMARS were developed specifically for evaluating mHealth apps that aim to improve consumers’ health. Meanwhile, menstrual apps were designed to help consumers keep track of their current health rather than improve it. Health apps must provide solutions customized to individual consumers [36]; reliability may be key in this respect [42]. This study’s results indicate that current health apps do not fully meet consumers’ requirements or desires with respect to content. To better identify consumer objectives and take account of them during the development of menstrual apps, a new evaluation scale is needed to evaluate menstrual apps.

### Limitations

This study had several limitations. First, the results cannot be generalized to all menstrual apps because a small number of evaluators evaluated only the most popular apps. Second, the apps were selected based on App Store searches with a limited timeframe. Updates to apps may result in differences between the analyzed content and that in the future. Third, the database is not an electronic database but the App Store. The App Store’s app recommendation function may have compromised an inconsistent search accuracy.

### Conclusions

In this study, consumer and health care provider ratings of menstrual apps were obtained using validated scales. Consumer preferred app had high scores of aesthetics and information, and evaluation scores differed between consumers and health care providers. The findings highlight the importance of consumer participation in menstrual app development and evaluation. This study is significant in that it is the first to compare health care providers’ and consumers’ menstrual app quality ratings. We expect our results to guide future mHealth app development and provide consumers with information on menstrual app content and quality. To provide high-quality apps for consumers, continuous quality evaluation research needs to be conducted, and the perspectives of both consumers and health care providers should be taken into account.

## Appendix

Multimedia Appendix 1 Mobile Application Rating Scale (MARS) and user version of the Mobile Application Rating Scale (uMARS) score of all 34 apps.

Multimedia Appendix 2 Content comparison between the top and bottom five apps (ranked according to the consumer and health care provider evaluation scores).

## Abbreviations

MARS: Mobile Application Rating Scale
mHealth: mobile health
OS: operating system
uMARS: user version of the Mobile Application Rating Scale
