# Supplementary material for: Menstrual Tracking Mobile App Review by Consumers and Health Care Providers: Quality Evaluations Study
Source: JMIR Mhealth Uhealth. 2023 Mar 1;11:e40921. doi: 10.2196/40921 (PMC10018377; doi:10.2196/40921)
Supplement: Multimedia Appendix 2 [file mhealth_v11i1e40921_app2.pdf]

Table 1. Content comparison between the top and bottom five apps base on uMARS

Content	uMARS Top 5 app					uMARS Bottom 5 app				
**Menstrual cycle management**										
Symptoms, pain	✓	✓	✓	✓	✓	✓	✓	✓		✓
Additional symptom	✓	✓	✓	✓	✓	✓	✓			✓
**Ovulation management**										
Calculate pregnancy probability										
Contraception methods						✓				
Both	✓	✓	✓	✓	✓			✓		✓
**Functions**										
Graphical chart		✓	✓	✓	✓	✓		✓		✓
Lock	✓	✓	✓	✓	✓	✓		✓		✓
Advice provision			✓							
Data export	✓	✓	✓	✓	✓	✓	✓	✓		
Predictions	✓	✓	✓	✓	✓	✓	✓	✓		✓
Login		✓	✓	✓	✓	✓	✓	✓		
**Education/knowledge**										
General health information			✓	✓				✓		
Personalized information										
Both										
Health screening										
**Sharing of information (with healthcare professional)**										
All information										
Only information specified by the consumer										
**Visualization**										
Menstruation or ovulation										
Menstrual cycle							✓	✓	✓	
All data	✓	✓	✓	✓	✓	✓				✓
**Notifications**										
Menstruation or fetility						✓				
Both	✓	✓	✓	✓	✓		✓	✓	✓	✓
Personlized alarms	✓		✓	✓	✓					
**Other features**										
Community										
Shopping										

Table 2. Content comparison between the top and bottom five apps base on MARS

Content	MARS Top 5 app					MARS Bottom 5 app				
**Menstrual cycle management**										
Symptoms, pain	✓	✓	✓	✓	✓		✓	✓		
Additional symptom	✓	✓	✓	✓	✓		✓	✓		
**Ovulation management**										
Calculate pregnancy probability									✓	
Contraception methods										
Both	✓	✓	✓	✓	✓			✓		
**Functions**										
Graphical chart		✓		✓	✓			✓		✓
Lock	✓	✓	✓	✓	✓			✓	✓	✓
Advice provision		✓						✓		
Data export	✓	✓	✓		✓		✓	✓		✓
Predictions	✓	✓	✓	✓	✓		✓	✓		✓
Login		✓		✓	✓		✓	✓		
**Education/knowledge**										
General health information					✓				✓	
Personalized information		✓								
Both										
Health screening		✓								
**Sharing of information (with healthcare professional)**										
All information		✓							✓	✓
Only information specified by the consumer					✓					
**Visualization**										
Menstruation or ovulation										
Menstrual cycle						✓	✓		✓	✓
All data	✓	✓	✓	✓	✓			✓		
**Notifications**										
Menstruation or fetility										✓
Both	✓	✓	✓	✓	✓	✓	✓	✓	✓	
Personlized alarms	✓	✓	✓	✓	✓			✓		
**Other features**										
Community		✓								
Shopping		✓			✓					
